# The role of day care in supporting older people living with long-term conditions

**DOI:** 10.1097/SPC.0000000000000391

**Published:** 2018-10-08

**Authors:** Catherine Lunt, Christopher Dowrick, Mari Lloyd-Williams

**Affiliations:** Academic Palliative and Supportive Care Studies Group (APSCSG), University of Liverpool, Brownlow Hill, Liverpool, England

**Keywords:** day care, long-term conditions, older people, outcome measures, volunteers

## Abstract

**Purpose of review:**

For older people with long-term conditions, regular structured activities within a community setting meeting others are thought to improve well being and quality of life. Historically local authority-run day care centres were widely available, but austerity measures have meant that in many areas, such provision has been markedly reduced and different models of day care services are being developed. There is little known about outcomes of day care provision for older people with long-term conditions.

**Recent findings:**

This review has critically examined the recent evidence on outcomes of day care provision for older people with long-term conditions and will focus on three areas – physical functioning, intergenerational provision and measurement of outcomes. In terms of interventions to improve physical functioning for older people with long-term conditions attending day care, there are few studies and it is difficult to generalize but there appears to be a trend for positive impact on physical functioning when activities are incorporated into a day care programme. There is a paucity of research on intergenerational provision, however, the small number of studies suggest positive benefits. Studies measuring outcomes for older people with long-term conditions attending day care services are very limited in terms of outcome data with the exception of a Canadian study, which suggested that attendance at day care could reduce hospital attendance and admissions.

**Summary:**

This review reveals a lack of research of day care provision for older people with long-term conditions. There is a suggestion in the small number of articles included in this review that there can be benefits both in terms of global outcomes of attendance and in improved physical functioning; there is limited evidence of the value of intergenerational provision. Robust research with collection of meaningful outcomes is required to ensure that the increasing number of older people with long-term conditions are enabled to access high-quality day care provision.

## INTRODUCTION

The definition of a long-term condition is a condition that cannot be cured but can be treated with medication. Long-term conditions include neurological diseases such as Parkinson's disease, multiple sclerosis, chronic obstructive pulmonary disease, heart failure, renal failure, and it is estimated that there are 1.8 million in the UK living with cancer and at least one long-term condition and further estimated that over 700 000 people are living with cancer in addition to three long-term conditions and the majority of these people will be older people.

There is a lack of robust evidence as to whether day care can improve the quality of life for older people living with long-term conditions. Little is known about different models of day care and their impact on the experience of those accessing services and whether some models of day care may demonstrate greater benefit, and therefore, be more appropriate for the future needs of older people with long-term conditions. An international systematic review by Fields *et al.*[1] found that the effectiveness of day care was difficult to assess because of the lack of a standardized definition. In addition, a number of studies explore the ‘service’ as opposed to the interventions offered within the day care provision. Although the respite function of day care is acknowledged and explored, less is known about other activities that may occur such as educational programmes or support groups. A systematic review of the effectiveness and cost effectiveness of different models of respite care for frail older people included day care as a form of respite and revealed limited evidence in terms of benefits beyond satisfaction amongst carers [2]. Day care is a neglected research area and there is a paucity of research into services and interventions, which could improve outcomes for multimorbidity across health and social care services [3,4]. In the UK, Age UK [5] undertook an evidence review exploring the effectiveness of day care in the UK and highlighted the paucity of research and indeed the quality of research. Day care service models are mixed [6] and can be provided by local authorities, private companies, independent, voluntary groups or a blend of more than one model. Frequently, the research undertaken in day care settings does not describe the populations or organizational structure making any findings difficult to interpret [7]. Day care models also vary internationally, and in many countries access to a day care service is dependent on the individual paying for the service. The regularity of day care has been highlighted as invaluable to families especially as other forms of respite, for example, overnight respite services can only be accessed at times of crisis and day care can provide regular intervention before crisis developed [8]. Zarit *et al.*[9] in a study from the USA found that family carers of a person with dementia who attended day care for minimum of 2 days a week for 3 months, benefited significantly more than those not accessing day care in terms of reducing caregiver depression and caregiver burden. There is evidence that day care improved coping mechanisms of family carers because of the respite function. Valadez *et al.*[10] found that carers felt less anxious and worried when they were not with their relative who attended day care, which in turn improved their relationship when they were together.

A recent systematic review from Korea [11] was conducted to identify what types of health interventions are effective and feasible for senior centre participants. The authors searched for health intervention studies conducted in senior centres published in English and Korean between 1990 and 2014. Of the 907 screened articles, 22 studies were selected. The review revealed that 59.1% of the interventions were provided by nurses and such health interventions resulted in positive effects on senior centre participants’ knowledge, health behaviours, clinical indices, and hospitalization rates, but few studies within the review reported on feasibility outcomes such as satisfaction and cost-effectiveness. The authors concluded that older people can access senior centres readily (however, this is dependent on country and location) but that health interventions and services within centres should be strengthened.

In this review, we will focus on recent articles published in the international literature regarding day care services for older people with long-term conditions including dementia and will focus on three areas – physical functioning, intergenerational provision and measurement of outcomes of attending day care facilities. As the number of articles are limited in this under researched area, we have included in this review, a search of articles from 2015 to the present day. 

**Box 1:**
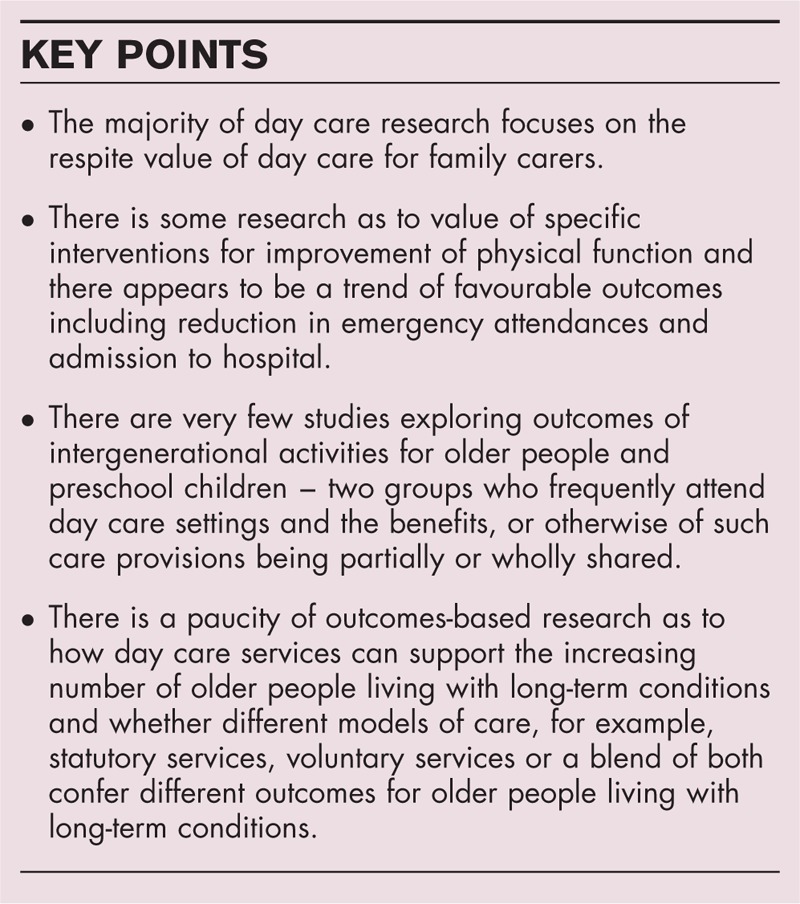
no caption available

## PHYSICAL FUNCTIONING

An article by Karania [12^▪▪^] reported a small pilot study to evaluate the impact of a bilaterally asymmetric gymnastics-based exercise programme on older people participating in a care home and day-centre setting located within the UK. A qualitative evaluation included observing sessions, interviews with older people participating, their spouses, family members and friends; interviews with staff; and a review of individual delivering the exercise programme. The pilot study found that older people participating in the programme showed a demonstrable improvement with aspects of their physical, emotional and cognitive ability and those with mild to advanced forms of dementia appeared to benefit the most. It was also reported that older people enjoyed the sessions and new friendships were developed. This study did not have a comparator group but provided useful data in benefits on asymmetric gymnastics-based exercise programme on older people, with varying levels of dementia.

Lin *et al.*[13] investigated the effects of Tai-Chi in conjunction with thera-band resistance exercise on functional fitness and muscle strength in community-based older people using a cluster randomized trial of 94 older people attending six senior day care centres in Taiwan. The group were assigned to thera-band resistance exercise or control group with the thera-band resistance exercise group receiving 60 min thera-band resistance exercise twice weekly for 16 weeks and the control group accessing routine activities in the day care centre but no Tai-Chi or resistance exercise. The results suggested that older people in the intervention group displayed a significant increase in muscle strength of upper and lower extremities and significant improvements were recorded on most measures of the Senior Fitness Test. The authors concluded that thera-band resistance exercise has the potential to improve functional fitness and muscle strength in community-based older people and can offer a well tolerated and appropriate form of physical activity, which staff can easily incorporate into the daily routine of older people in day care centres.

Park *et al.*[14^▪▪^] trialled the effects of the Sit ‘N’ Fit Chair Yoga, compared with a Health Education program (HEP), on pain and physical function in older adults with lower extremity osteoarthritis, who could not participate in standing exercise. A two-arm randomized controlled trial located in an older people residential facility and day care centre in the USA. One hundred and thirty-one older people were randomly assigned to chair yoga or health education programme. Thirteen were lost after assignment but prior to the intervention; six were lost to follow-up during the intervention; 106 of 112 completed at least 12 of 16 sessions (95% retention rate). Older people in the trial attended either chair yoga or HEP within twice-weekly 45-min sessions for 8 weeks. Measures were collected regarding pain, pain interference; balance, gait speed, fatigue, functional ability and measured at baseline, after 4 weeks of intervention, at the end of the 8-week intervention and postintervention (1 and 3 months). The chair yoga group had a significantly greater reduction in pain interference during the intervention (*P* = 0.01), which was sustained at 3 months. Gait speed and fatigue were improved in the yoga group during the intervention but not sustained postintervention. Chair yoga had no effect on balance. The authors concluded that an 8-week chair yoga program was associated with reduction in pain, pain interference, and fatigue, and improvement in gait speed, but only the effects on pain interference were sustained 3 months postintervention.

## INTERGENERATIONAL

There is a tendency in Western society for generations to remain separate – in other cultures, intergenerational activities occur as a natural part of family life as three and sometimes four generations of families including extended family networks live together or in close proximity [15]. There is currently a great deal of interest in intergenerational activities within older people care facilities including day care with such sessions being documented within Television documentaries [16]. We were only able to identify two articles within the time frame of this review, one relating to preschool children and one to older children/teenagers.

An article from Australia [17] and studied an innovative model of older people, carers (parents, grandparents or nannies) and children aged 0–4 years old. The objective of the study was to explore the benefits of participating in an intergenerational playgroup program (IPP) within an older people care facility for all participants. Older people were invited to complete SF36 and the only notable result was a trend for decline in energy and fatigue, and there were no differences in the Geriatric Depression Scale. Qualitative interviews reported positive findings from both older people and carers of children, of the value of the project including intergenerational experiences, two-way contributions, friendships, personal growth, and environmental considerations, for example, access to outdoor areas. The authors reported that the playgroup within an older people's care facility, for older people, including those with dementia, provided meaningful and fun activities across the generations and created friendships and connections for children, their carers and older people.

We identified one article that explored intergenerational care from a perspective of consumer preferences and whether parents were willing to pay and pay more for provision of such care. Two models of intergenerational care were identified – a shared campus where older people day care facilities and children's facilities are co-located and visiting model, where children visit older people (or vice versa). Key attributes of both models were identified as respite day care; a common educational pedagogy across generations; screening; monitoring and evaluation of participant outcomes. Parents were more likely to wish for intergenerational care and carers of older people demonstrated a higher willingness to pay for such care. The authors report that there is a demand within the Australian community for intergenerational care.

A study by Spiteri [18] analyzed the motivators for older people to attend ‘day centres’ in Malta and what older people believed they gained from young people aged 16–17, attending a vocational college in Malta and studying health and social care, carried out placements within day centres. Focus groups revealed that the main findings were that older people believed the students were helping them on an emotional level by providing encouragement, and on a practical level, by offering insights that helped with modern-day life.

## OUTCOMES OF ATTENDING A DAY CARE FACILITY

The effectiveness of day care was difficult to assess because of the lack of a standardized definition [1] and the lack of a strong conceptual model means that there is a difficulty connecting participant needs and services to specific outcomes. This causes difficulty in understanding what works, for whom and in what circumstances within the day care setting [19]. A study by Kelly [20^▪▪^] investigated the effect of day programme attendance by older people living at home on numbers and rates of emergency room admissions, hospital admissions and days in hospital. This large study matched older people attending an adult day programme with a nonattendee on the basis of similar propensity scores from clinical, psychosocial and functional co-variates. Analysis revealed significantly lower mean 100-day rates of emergency room registrations/attendances, hospital admissions and days in hospitals for attendees, compared with matched nonattendees. The author concluded that lower rates were largely attributable to longer stays in the home care program for attendees, however, the mean number of days in hospital was still significantly lower compared with nonattendees. The authors report that these findings replicate and extend results from previous research that reported a decreased reliance on costly healthcare services by older people who attend adult day programs.

A review of day care centres for people with dementia and the impact on their family carers was carried out in an article from Norway by Tretteteiga *et al.*[21]. The integrative review including 19 studies was used: qualitative [2], quantitative nonrandomized [8], quantitative descriptive studies [7] and with mixed-method design [2]. The review revealed that family carers experienced day care as a respite service, and also a support service, improving their competence in caring. Quality of the day care influenced its use as did the individual needs and behavioural issues of the person with dementia. The article suggested that day centres can reduce burden for family caregivers and help in their caring role.

There is debate as to whether people with dementia should attend a Dementia Specific day centre or a generic service. A study from Northern Ireland exploring experiences of staff working in generic day care centres of supporting people living with dementia was carried out by Laird *et al.*[22^▪▪^]. The qualitative study utilized focus groups and staff reported that a generic day care service that provides an integrated care and social support to older people irrespective of whether they have or have not dementia, is realistic and manageable. The article concluded that regular attendance at a day care centre may support older people with dementia and their families. An on line evidence brief [23^▪▪^] reported the findings of research of 4 day centres with 23 older people;10 family members; 23 staff and volunteers and 13 local authority staff interviewed. The study reported that older people gained an opportunity to contribute and a sense of purpose in attending day centres and that day centres may particularly benefit people over 85, who may find difficulty accessing generic community activities.

## CONCLUSION

Improved healthcare and more effective treatments mean that more older people with long-term conditions are living longer than ever before.

People with multiple long-term conditions constitute 30% of the population and account for 70% of the total spend of UK healthcare budget. The CQC reported that joined up social care and health across England would save £2 billion just with regards to multiple admissions of those aged 75 and over [24]. For many older people who are living with multiple long-term conditions, the sense of isolation is huge and opportunities to engage with the wider community are limited and they are less engaged with the community. Day care can enable participation, interaction with peers and a sense of contribution to the community [25–27]. This review has revealed there are a dearth of articles exploring impact of day care services on older people with long-term conditions and where there are studies, many are descriptive and seldom determine impact of day care in terms of outcomes for older people. Improving physical function is known to improve physical health for older people but little research has been carried out as to whether day care services can provide such interventions, and if so the effectiveness on such interventions on reducing morbidity. As a western society, age groups are frequently segregated and opportunity for regular contact between the old and the young is limited. As the very old and the very young frequently attend ‘day care,’ it is surprising that such little attention has been paid as to how the two age groups could receive care under the same roof with mutual reciprocity. Research with older people with long-term conditions is difficult because of their frailty; however, we need to ensure that all care provided, including care that is seen as being predominantly social, is evaluated and rigorously researched with collection of meaningful outcomes, if we are to ensure that the increasing number of older people living with long-term conditions receive the best possible care.

## Acknowledgements

We are grateful to members of the Academic Palliative and Supportive Care Studies Group (APSCSG) at the University of Liverpool for their support with this review.

This review was supported by the National Institute for Health Research (NIHR) Collaboration for Leadership in Applied Health Research and Care North West Coast (NIHR CLAHRC NWC). The views expressed are those of the authors and not necessarily those of the NHS, NIHR or Department of Health.

### Financial support and sponsorship

None.

### Conflicts of interest

There are no conflicts of interest.

## REFERENCES AND RECOMMENDED READING

Papers of particular interest, published within the annual period of review, have been highlighted as:

▪ of special interest
▪▪ of outstanding interest

## References

[1] Fields NL Anderson KA Dabelko-Schoeny H The effectiveness of adult day services for older adults: a review of the literature from 2000-2011. J Appl Gerontol 2014; 33:130–163. 24652952 10.1177/0733464812443308

[2] Mason A Weatherly H Spilsbury K< ET-AL> A systematic review of the effectiveness of different models of community-based respite care for frail older people and their carers. Health Technol Assess 2007; 11:1–157. 10.3310/hta11150 17459263

[3] Molzahn AE Gallagher E McNulty V Quality of life associated with adult day centers. J Gerontol Nurs 2009; 34:37–46. 10.3928/00989134-20090706-02 19681562

[4] McVicker H Defining daycare. Belfast Social Services Inspectorate. Available at: https://www.kcl.ac.uk/sspp/policy-institute/scwru/people/orellana/Day-centres-for-older-people-Briefing-2017.pdf 2004.

[5] Smith SM Wallace E O’Dowd T Fortin M Interventions for Improving outcomes in patients with multimorbidity in primary care and community settings (Review). Cochrane Collaboration [online]. Available at: http://www.thecochranelibrary.com [Accessed 2 April 2015] 10.1002/14651858.CD006560.pub4 PMC8092473 33448337

[6] Age UK. Effectiveness of day services Summary of research evidence. Age UK. 2011.

[7] Gridley K Good support for people with complex needs: what does it look like and where is the evidence. NIHR.

[8] Manthorpe J Moriarty J Examining day centre provision for older people in the UK using the Equality Act 2010: findings of a scoping review. Health Social Care Community 2014; 22:352–360. 10.1111/hsc.12065 23952653

[9] Zarit SH Stephens MA Townsend A Greene R Stress reduction for family caregivers: effects of adult day care use. J Gerontol B Psychol Sci Soc Sci 1998; 53:S267–S277. 9750575 10.1093/geronb/53b.5.s267

[10] Valadez AA Lumadue CA Gutierrez B De Vries S Family caregivers of impoverished Mexican American elderly women: the perceived impact of adult day centers. Family Soc 2005; 86:384–392.

[11] Song M Seo K Choi S Seniors centre-based health intervention programmes in the United States and South Korea: A systematic review. Int J Nurs Pract 2017; 23: doi:10.1111/ijn.12568. 10.1111/ijn.12568 28691357

[12▪▪] Karania VK Evaluation of age & dementia friendly gymnastics programme. Working with Older People 2017; Available at: https://www.ageuk.org.uk/globalassets/age-uk/documents/reports-and-publications/evaluation-reports/bgf_pilot_evaluation_report.pdf. [Accessed 13 September 2018].

[13] Lin S-F Sung H-C Li T-L The effects of Tai-Chi in conjunction with thera-band resistance exercise on functional fitness and muscle strength among community-based older people. J Clin Nurs 2015; 24:1357–1366. 25620554 10.1111/jocn.12751

[14▪▪] Park J McCaffrey R Newman D A pilot randomized controlled trial of the effects of chair yoga on pain and physical function among community-dwelling older adults with lower extremity osteoarthritis. J Am Geriatr Soc 2017; 65:592–597. 28008603 10.1111/jgs.14717 PMC5357158

[15] Lien YF Huang HM Challenges in intergenerational caregiving for frail older people: a multiple case study. Nurs Health Sci 2017; 19:81–87. 28026105 10.1111/nhs.12314

[16] Channel4. Old people's homes for 4 year olds. Available at: http://www.channel4.com/programmes/old-peoples-home-for-4-year-olds.

[17] Skropeta CM Colvin A Sladen S An evaluative study of the benefits of participating in intergenerational playgroups in aged care for older people. BMC Geriatr 2014; 14:109. 25292218 10.1186/1471-2318-14-109 PMC4197292

[18] Spiteri D What do older people learn from young people? Intergenerational learning in ‘day centre’ community settings in Malta. Int J Lifelong Educ 2016; 35:235–253.

[19] Dabelko-Schoeny H King S In their own words: participants’ perceptions of the impact of Adult Day services. J Gerontol Social Work 2010; 53:176–192. 10.1080/01634370903475936 20094936

[20▪▪] Kelly R The effect of adult day program attendance on emergency room registrations, hospital admissions, and days in hospital: a propensity-matching study. Gerontologist 2017; 57:552–562. 26640154 10.1093/geront/gnv145

[21] Tretteteig S Vatne S Rokstad AM The influence of day care centres designed for people with dementia on family caregivers - a qualitative study. BMC Geriatr 2017; 17:5. 28056843 10.1186/s12877-016-0403-2 PMC5216603

[22▪▪] Laird EA McGurk P Reid B Ryan A Making the best of what we have’: the lived experiences of community psychiatric nurses, day centre managers and social workers supporting clients with dementia attending a generic day care service. Int J Older People Nurs 2017; 12:10.1111/opn.12157 28660749

[23▪▪] Orellana. Available at: https://www.kcl.ac.uk/sspp/policy-institute/scwru/people/orellana/Day-centres-for-older-people-Briefing-2017.pdf.

[24] CQC. People need health and social care services that are more joined-up and person-centred, says CQC. 2010. Available at: http://www.cqc.org.uk/content/people-need-health-and-social-care-services-are-more-joined-and-person-centred-says-cqc [Accessed 19 March 2015]

[25] Embrey N Exploring the lived experience of palliative care for people with MS: therapeutic interventions. J Neurosci Nurs 2009; 5:311–317.

[26] Embrey N Exploring the lived experience of palliative care for people with MS,3: views of group support. J Neurosci Nurs 2009; 5:403–408.

[27] Garcia-Martin MA Gomez Jacinto L Martimportuges-Goyenechea A structural model of the effects of organized leisure activities on the well being of elder adults in Spain. Act Adapt Aging 2015; 28:19–34.

